# P-268. Root Cause Analysis of Healthcare-Associated Infections: A Case Study Methodology Using Pareto Charts, Ishikawa Diagrams, and Machine Learning

**DOI:** 10.1093/ofid/ofae631.472

**Published:** 2025-01-29

**Authors:** Bráulio R G M Couto, Maria Clara Bueno, Louranny Cristina Góis, Estevão Urbano Silva, Virginia Andrade, Ana Beatriz Silva, Joel T Oliveira, Erika Vrandecic

**Affiliations:** AMECI – Associação Mineira de Epidemiologia e Controle de Infecções, Belo Horizonte, Minas Gerais, Brazil; Biocor Instituto – Rede D’Or, Belo Horizonte, Minas Gerais, Brazil; Biocor Instituto – Rede D’Or, Belo Horizonte, Minas Gerais, Brazil; Hospital Madre Teresa, Belo Horizonte, Minas Gerais, Brazil; Hospital Madre Teresa, Belo Horizonte, Minas Gerais, Brazil; Biocor Instituto – Rede D’Or, Belo Horizonte, Minas Gerais, Brazil; Biocor Instituto, Nova Lima, Minas Gerais, Brazil; Biocor Instituto – Rede D’Or, Belo Horizonte, Minas Gerais, Brazil

## Abstract

**Background:**

This paper introduces a method for analyzing the root causes of healthcare-associated infections (HAIs), stressing the importance of a holistic approach that merges clinical and epidemiological data.

**Figure 1:**
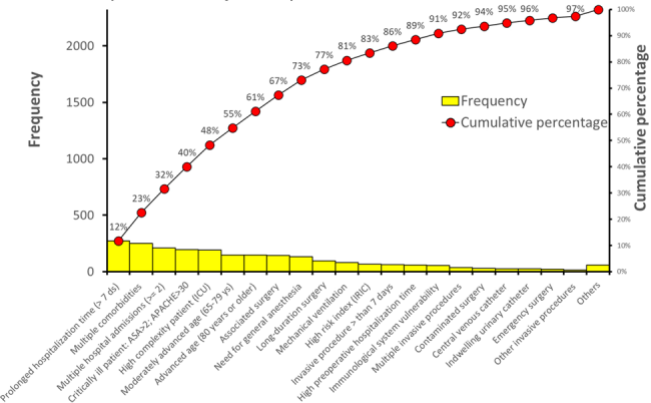
Pareto Chart with the Main Specific Secondary Factors for HAIs Diagnosed in a General Hospital, January to September 2023.

Pareto Chart with the Main Specific Secondary Factors for HAIs Diagnosed in a General Hospital, January to September 2023.

**Methods:**

Risk and causal factors for HAI were identified and categorized into primary and secondary groups. A review of case studies and literature identified 63 factors, which were then organized into four primary categories: surgical factors, invasive procedures, care process failures, and intrinsic patient characteristics. Each category included specific secondary factors; for instance, intrinsic patient characteristics encompassed 18 factors such as neurogenic bladder, diabetes mellitus, and obesity. Active infection surveillance involved recording these factors for each patient and correlating them to specific infections. The analysis utilized Pareto charts, Ishikawa diagrams, and decision trees to summarize root causes in a case series study. From Pareto, construct Ishikawa, global (HAI), and for each topography.

**Figure 2:**
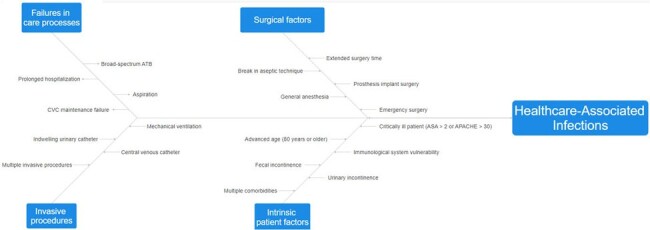
Ishikawa Diagram for HAIs Diagnosed in a General Hospital, January to September 2023.

Ishikawa Diagram for HAIs Diagnosed in a General Hospital, January to September 2023.

**Results:**

During the active surveillance of 386 healthcare-associated infections (HAIs) diagnosed in a general hospital from January to September 2023: tracheobronchitis and pneumonia (178), surgical site infection (74), urinary tract infection (56), primary bloodstream infection (29), gastrointestinal infection (21), and other infections (28). A total of 2,323 potential risk and causal factors were recorded. The number of factors associated with each infection varied, ranging from zero (indicating no factors were identified with the infection) to 18 factors identified in a single case. The primary factor that predominated was intrinsic patient factors (44%), followed by failures in care processes (27%), surgical factors (21%), and invasive procedures (8%). With the risk factor data recorded during active infection surveillance, it's feasible to automate case-by-case critical analysis, for example, through mail merges, integrating data in spreadsheets with texts in Word.

**Figure 3:**
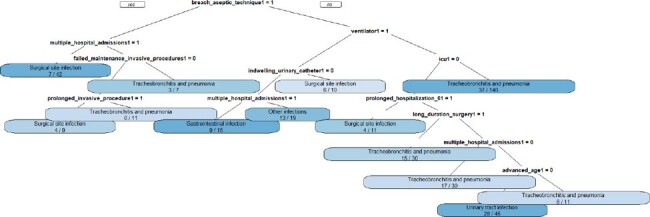
Decision Tree Based on Secondary Factors to Stratify the Type of HAI.

Decision Tree Based on Secondary Factors to Stratify the Type of HAI.

**Conclusion:**

Our analysis illustrates how integrating clinical and epidemiological data can pinpoint critical factors contributing to HAIs. In addition to case series analysis, recording factors during active surveillance automates critical case-by-case analysis.

**Figure 4:**
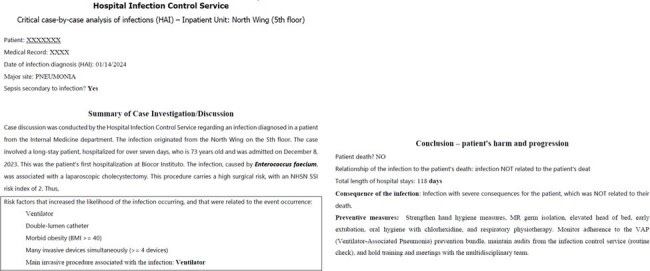
Example of an automated critical case-by-case analysis of a ventilator-associated pneumonia.

Example of an automated critical case-by-case analysis of a ventilator-associated pneumonia.

**Disclosures:**

**All Authors**: No reported disclosures

